# Supplementary material for: Western Kenyan Anopheles gambiae showing intense permethrin resistance harbour distinct microbiota
Source: Malar J. 2021 Feb 8;20:77. doi: 10.1186/s12936-021-03606-4 (PMC7869237; doi:10.1186/s12936-021-03606-4)

Suppl. 2: Shannon diversity indices showed no significant difference in diversity of bacterial taxa between individual resistant (n = 39) and susceptible (n = 36) *An. gambiae s. s.* (H= 0.45, *p*= 0.50). Comparisons were performed using Kruskal-Wallis tests (H) with Benjamini–Hochberg FDR correction (q-value). Significance was determined at q<0.05


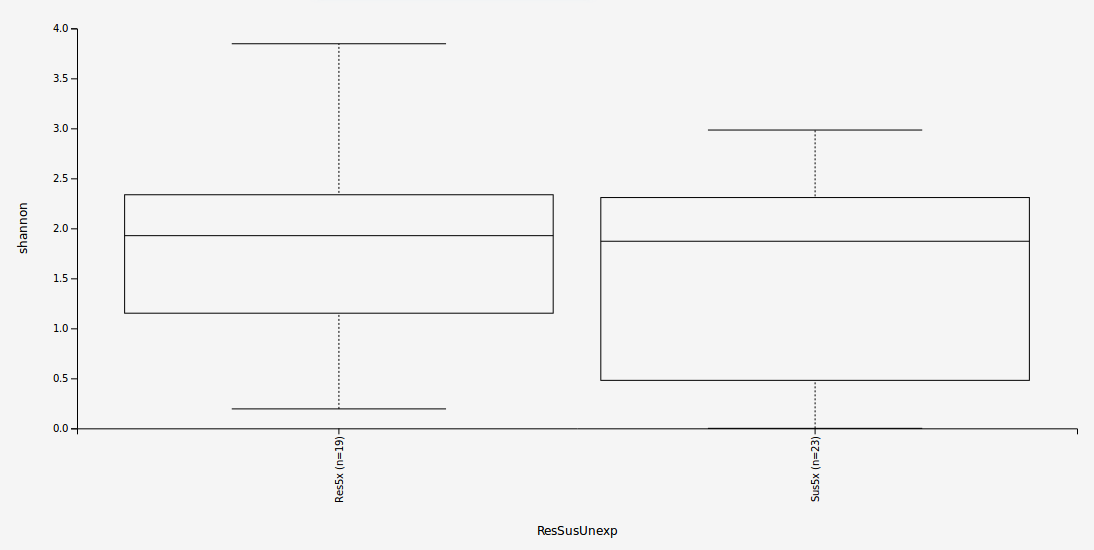

Supplement: Supplementary file 2 — Additional file 2. Shannon diversity indices. [file 12936_2021_3606_MOESM2_ESM.docx]
