# Supplementary material for: Western Kenyan Anopheles gambiae showing intense permethrin resistance harbour distinct microbiota
Source: Malar J. 2021 Feb 8;20:77. doi: 10.1186/s12936-021-03606-4 (PMC7869237; doi:10.1186/s12936-021-03606-4)

Suppl. 6: Shannon alpha diversity index rarefaction curves

The number of ASVs from the microbiota of individual permethrin resistant (n = 39) and susceptible (n = 36) *An. gambiae s. s.*, along with the depth at which rarefaction was performed (100 ASVs per sample). The rarefaction plots show average Shannon diversity index and range (boxplots: minimum, median and mean) for 10 iterations of Shannon diversity analysis. The rarefaction plots plateau before the depth of rarefaction, indicating that increasing sequencing depth resulted in no/negligible change in Shannon indices.


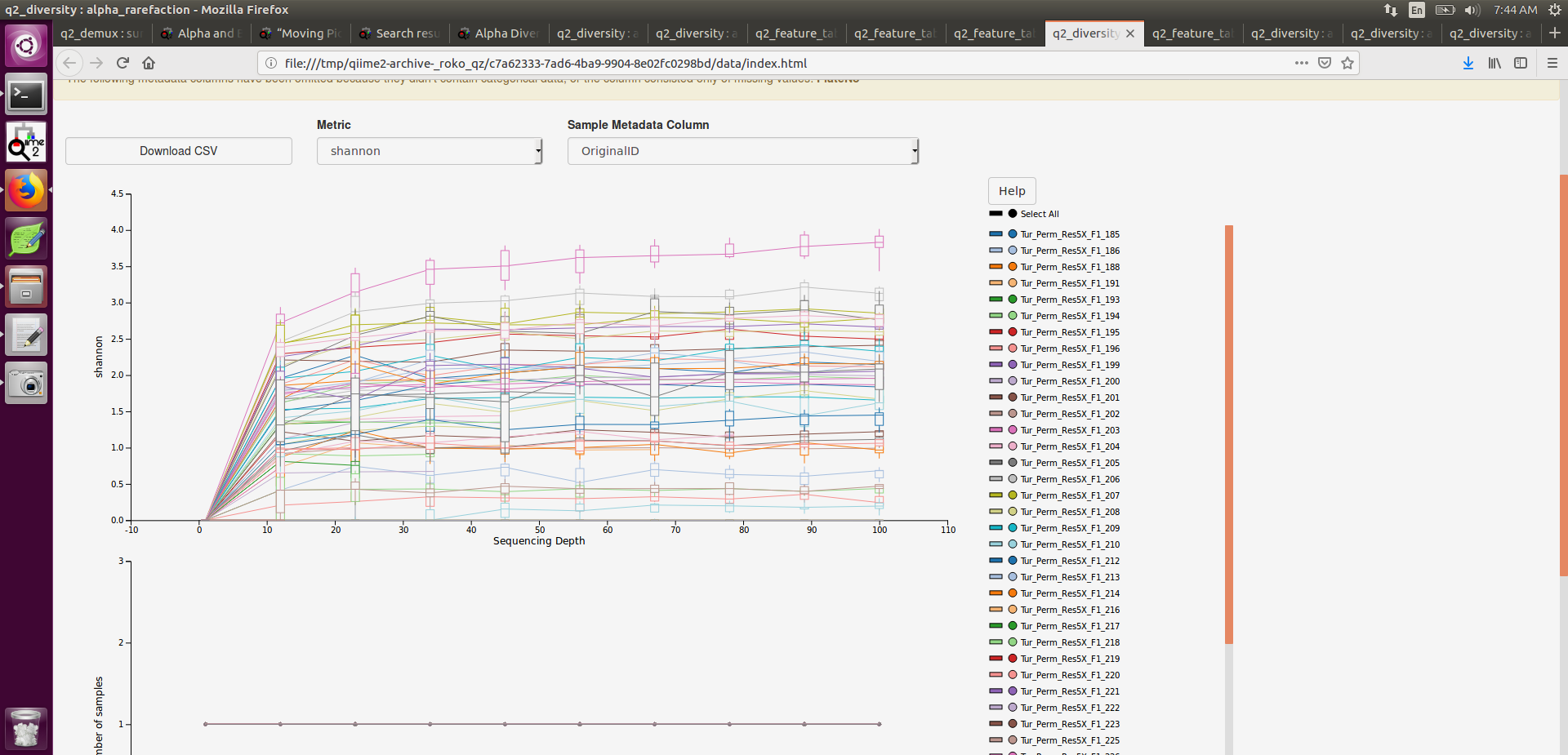

Supplement: Supplementary file 6 — Additional file 6. Shannon alpha diversity index rarefaction curves. [file 12936_2021_3606_MOESM6_ESM.docx]
